# Effect of a dietary and exercise intervention in women with overweight and obesity undergoing fertility treatments: protocol for a randomized controlled trial

**DOI:** 10.1186/s40795-021-00454-y

**Published:** 2021-08-17

**Authors:** Kindann Fawcett, Audrey Martinez, Meghan Crimmins, Clark Sims, Elisabet Børsheim, Aline Andres

**Affiliations:** 1 Arkansas Children’s Nutrition Center, 15 Children’s Way, Slot 512-20B, Little Rock, AR 72202 USA; 2 Department of Pediatrics, University of Arkansas for Medical Sciences, Little Rock, AR USA; 3 Arkansas Children’s Research Institute, Little Rock, AR USA

**Keywords:** Obesity, Mediterranean diet, Exercise, Peri-conception, Oocyte, Follicular fluid

## Abstract

**Background:**

Distinct molecular, inflammatory, and metabolic signatures are present in oocytes and follicular fluid derived from women with obesity when compared to those derived from normal weight women, which suggest existing signals that may program future offspring for metabolic diseases. This study aims to assess the feasibility and efficacy of a peri-conception nutrition and exercise intervention on mitigating obesity-associated changes in oocyte gene expression profiles and follicular fluid metabolites.

**Methods:**

This single blinded randomized control trial will include 120 women with a BMI of 25–45 kg/m^2^, ≥21 years of age, and undergoing in vitro fertilization (IVF) treatments. Participants will be randomized to standard of care (*N* = 60) or an intervention group (*N* = 60) in a block design by polycystic ovary syndrome status. The intervention will combine a dietary component (Mediterranean meal plan) with exercise prescription following the Physical Activity Guidelines for Americans. Participants will be assessed pre- and post-intervention. The standard of care group will be offered to join the intervention group if the IVF treatments are unsuccessful as a cross over design. Recruitment is anticipated to start in July of 2021. Primary outcomes will include single oocyte gene expression profiles and follicular fluid metabolites. Mann-Whitney U nonparametric tests will be used to assess potential differences for each stratum. Follicular fluid and serum metabolites will be analyzed using a one-factor Analysis of Covariance (ANCOVA) at four levels, pair-wise comparisons using Tukey-Kramer post-hoc tests will be used to identify groups whose means differ significantly while retaining the family-wise error rate at 5%. When the design is balanced, two-way Analysis of Variance (ANOVA), or non-parametric Friedman test will be used in data analysis. Additionally, general linear models and ANCOVA may be used to control for covariates. Significance will be set at *p* < 0.05. Findings will be disseminated via peer-reviewed manuscripts and presentations at scientific conferences.

**Discussion:**

This study will provide novel data and key information on the impact of a dietary and exercise intervention on oocyte gene expression and follicular fluid content. Results will demonstrate the potential of such intervention in mitigating obesity-induced changes in oocyte gene expression and follicular fluid metabolites.

**Trial registration:**

ClinicalTrials.gov (NCT04273048): submitted November 13, 2019; posted February 17, 2020.

## Background

Because of obesity’s increasing prevalence and limited treatment success, one approach to stem the obesity epidemic involves reducing the risk of obesity as early in life as possible. The Developmental Origins of Health and Disease (DOHaD) hypothesis suggests there are critical windows of development and growth, such as the in utero environment, that may significantly impact health for years to come through metabolic programing [1]. Undernutrition during this time alters metabolic tissues and systems in ways that confers vulnerability to cardio-metabolic diseases such as metabolic syndrome [2]. Research demonstrates that peripheral organ development is adversely affected by maternal obesity, with sex-specific changes in metabolic, cardiovascular, immune, and microbiome physiology in offspring, which may be transmitted through placental adaptations for lipid accumulation, altered nutrient transport, inflammation, and oxidative stress [3]. Similarly, other research involving experimental animals focused on manipulating the maternal diet during pregnancy and impairing fetal nutrition showed permanent changes in tissue structure, body composition, endocrine responses and metabolism in the offspring and a vulnerability to adult hypertension and diabetes [4]. Epidemiological analyses demonstrated that lower birth weight is a risk indicator for adult cardiovascular disease, hypertension, type 2 diabetes and metabolic syndrome (cardio-metabolic disease) [1]. Maternal obesity during pregnancy confers a major risk to offspring obesity [5], but less is known about the impact of peri-conception factors that influence metabolic programming. This research evolved into the concept of metabolic programming, where early periods of development have critical impact on multiple organ systems and lead to vulnerability to cardio-metabolic disease [2].

We have reported distinct molecular, inflammatory, and metabolic signatures in single oocytes and follicular fluid derived from obese women when compared to normal weight women preparing to undergo in vitro fertilization (IVF) [6]. The molecular disturbances found in oocytes of overweight women could be mitigated through an intervention involving a healthier diet and increased physical activity (PA) during the peri-conception period [7–9].

Models of diet-induced obesity in mice have shown that exercise improves, but does not reverse, damage imparted on oocytes as a result of a high fat diet [10]. A systematic review and meta-analysis of human studies found that exercise improved pregnancy rates (risk ratio 2.10, 95% CI: 1.32–3.35) in women with reproductive health problems like polycystic ovary syndrome (PCOS) [11]. Studies have also demonstrated promising maternal-fetal benefits of exercise during pregnancy such as improved physical fitness [12], more appropriate gestational weight gain [13], a decreased risk for preeclampsia and other pregnancy-specific diseases like gestational diabetes [14, 15], and decreased odds of macrosomia at birth without affecting the odds of growth-restricted, preterm, or low birth weight babies [12, 16, 17]. Studies have demonstrated that adherence to a Mediterranean diet, characterized by higher intake of unsaturated fats, lower intake of animal fats, and lower ratios of omega-6 to omega-3 fatty acids [18], is also associated with a reduced risk of many chronic diseases [19], improved fertility rates [odds ratio 1.4, 95% CI: 1.0–1.9] [20], as well as larger embryo yield in IVF [21], and better pregnancy outcomes [7, 20, 22, 23].

Despite the evidence of a healthier diet improving IVF procedure outcomes, there is little evidence on the molecular changes at the level of the oocyte or follicular fluid that occur due to alterations in diet, and virtually nothing is known about the impact of the combined effect of healthy diet and increased PA. Thus, we have designed a study with the primary objective of assessing the efficacy of a peri-conception nutrition and exercise intervention on mitigating obesity-associated changes in oocyte gene expression profiles and follicular fluid metabolites. Secondary objectives include determining the effect of the intervention on cumulus cells gene expression as well as the feasibility and acceptability of the intervention (using a standardized taxonomy [24]). To help address these objectives, overall metabolic health indices such as fitness, blood pressure, insulin sensitivity, triglycerides, C-reactive protein and interleukin-6 concentrations will be assessed.

## Methods

**Fig. 1:**
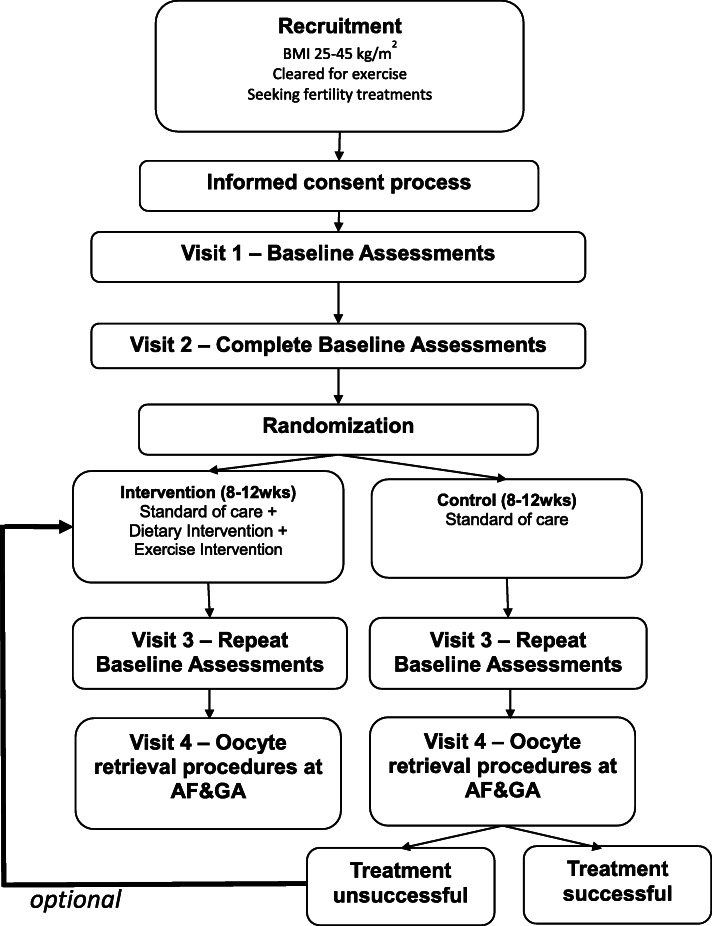
Study Flow Chart. Protocol schematic diagram including informed consent, visits, randomization, intervention and control groups, fertility clinic procedures, and cross over design. AF&GA: Arkansas Fertility and Gynecology Associates, BMI: Body Mass Index

This is a single-blinded randomized control trial testing the efficacy of a dietary and exercise intervention for women with overweight and obesity undergoing infertility treatment. Ethical approval was obtained from the UAMS Institutional Review Board (UAMS IRB-260113), version 10 of the protocol. This article follows the SPIRIT (Standard Protocol Items: Recommendations for International Trials) guidelines for reporting clinical trial protocols [25]. Table 1 and Fig. 1 detail the time schedule of enrollment, interventions, and assessments. Findings will be disseminated via peer-reviewed manuscripts and presentations at scientific conferences, at which time protocol amendments will be discussed.

**Table 1:** Schedule of enrollment, interventions, and assessments at specific time points

			Study Period			
	Staff Member	Pre-study Screening/ Consent	Baseline	Allocation		Post-Allocation
**Time Point**			Visit 1	Visit 2	Visit 3	Retrieval
RECRUITMENT/ENROLLMENT						
Eligibility Screen	Research Team Member	x				
Medical Record Release and Medical Release for Exercise	Medical Provider/Arkansas Fertility and Gynecology	x				
Informed Consent	Research Team Member		x			
Allocation of Treatment Groups	Clinical Coordinator or PI			x		
Compensation	Research Team Member		x		x	x
INTERVENTION						
Diet, Exercise, Coaching*	Research Team Member		x	x^1^	x^1^	x
Standard of Care	Arkansas Fertility and Gynecology		x	x	x	x
ASSESSMENTS						
Demographic and Contact Information	Research Team Member		x		x	
Medical History	Research Team Member		x		x	
Vitals and Anthropometrics	Research Team Member		x		x	
Fasted blood collection	Phlebotomist		x		x	
Urine sample collection	Participant		x		x	
Stool sample collection	Participant		x		x	
Body Composition	Research Team Member		x		x	
Resting Energy Expenditure	Research Team Member		x		x	
Activity Energy Expenditure	Participant Worn Monitor		x		x	
Fitness Testing	Exercise Physiologist		x		x	
3 Day Food Records	Nutritionist		x			
Coaching	Research Team Member			x^1^	x^1^	
Physical Activity Questionnaire	Participant Self-Assessment		x		x	
Cohen’s Perceived Stress Scale	Participant Self-Assessment		x		x	
Beck Depression Inventory-II	Participant Self-Assessment		x		x	
Oocyte and Follicular Fluid Retrieval	Arkansas Fertility and Gynecology					x
Hormonal Panel and Birth Outcomes	Arkansas Fertility and Gynecology					x
Feasibility Qualitative Interviews	Research Team Member					x^1^

x- All Participants, x^1^-Only the intervention group, * Diet, Exercise, and Coaching will take place after Visit 2 of study, intervention will last 8–12 weeks depending on oocyte retrieval process

### Study setting

This multi-component intervention will be conducted primarily via telemedicine, thus the location will vary and may include places like the participants’ homes or a local gym. Participants will be undergoing fertility treatments at Arkansas Fertility and Gynecology Associates (AF&GA), which serves Arkansas and surrounding states. Data collection will take place within AF&GA and the Arkansas Children’s Nutrition Center (ACNC), both of which are located in Little Rock, Arkansas.

### Eligibility and recruitment

Criteria for study inclusion are women with a BMI between 25 and 45 kg/m^2^, age of 21 years or over, and planning to undergo in vitro fertility treatment. Participants reporting the following will be excluded from the study: pre-existing conditions (e.g. sexual transmitted diseases) that will affect the outcomes of the study as determined by the principal investigator; current use of recreational drugs, tobacco, nicotine, or alcohol; food allergies, food intolerances, or food preferences which would interfere with compliance to the meal plan; and contraindications to exercise or already meeting the Physical Activity Guidelines for Americans [8] (150 min moderate activity/week or 75 min of vigorous activity/week with resistance exercise on 2 or more days/week.

Recruitment of participants will be conducted by the AF&GA team using IRB-approved tools. Advertisements and information sheets will be distributed to eligible participants electing to undergo fertility treatments. Women who are interested in participating in the study can inform the AF&GA team, call the ACNC recruitment line, send an e-mail expressing their interest, or express their interest through the ACNC website. To establish eligibility for participation in the study, a standardized questionnaire will be administered by phone.

### Consent and randomization

The informed consent process will educate potential participants about the research study through IRB-approved methods. Eligible participants will be contacted, and a copy of the informed consent will be provided to the participant prior to signing consent documents. A research team member will reach out to potential participants 2 days prior to the scheduled visit to perform a COVID-19 screening and answer questions on the intervention components of the study. At the first visit, the research team will review all study procedures and the informed consent documents with the potential participant. Participants will be given sufficient time to review the consent form, ask questions, and receive clarification prior to signing any documents. If eligible and interested in enrollment, informed consent will be obtained from the participants and documented.

Women will be randomized, with computer generated randomization, to a standard of care group (*N* = 60) and an intervention group (*N* = 60). Due to the high incidence of obese women with Polycystic Ovarian Syndrome (15–30%) [26], compared with other infertility diagnoses, women will first be classified into one of two strata based on the presence or absence of PCOS. Within each stratum, the proposed study will randomize the enrolled participants into either the intervention group (*N* = 30) or the standard of care group (*N* = 30). Thus, the potential for confounders of infertility, besides PCOS, should be equally distributed between the groups and not affecting the main outcome of interests. The study will have four experimental arms: PCOS-standard of care, PCOS-intervention, no PCOS-standard of care, no PCOS-intervention. Allocation will happen at the second visit (after consent and the collection of baseline measures) by providing the participant with an opaque, sealed envelope containing their allocation.

### Interventions

The intervention, combining an exercise and a dietary component along with the standard practice from AF&GA, will take place during the weeks leading up to oocyte retrieval and continue until 2 weeks after implantation. We expect the intervention to last 8–12 weeks, depending on the participant’s specific fertility treatment plan.

The intervention will be delivered by trained research team members who will meet with participants twice weekly and serve as their health coach in order to track compliance and support adherence to the meal and exercise plan. This contact will occur via their preferred contact method (e.g., telephone, text messages, email, or using applications such as FaceTime). All interventionists will complete an intensive training on the intervention protocol and motivational interviewing techniques [9, 27]. They will be provided with detailed counselor guides to follow. Prior to conducting a coaching session with a participant, they will be required to be certified by one of the study investigators in providing both initial and subsequent intervention sessions. All sessions will be video or audio recorded; 15% of sessions will be randomly selected, reviewed, and scored on a fidelity rating scale to ensure that the protocol is being implemented as intended. Fidelity ratings will include building and maintaining rapport; discussion of daily meal plan adherence and compliance; discussion of weekly exercise adherence and compliance [28]; use of SMART goal setting techniques [29]; engagement with participant in problem solving as needed; and use of reflective listening, positive reinforcement, and summarizing techniques [30]. Feedback will be provided to the coach on every session reviewed.

#### Exercise intervention

The exercise intervention will be based on the Physical Activity Guidelines for Americans for exercise prescription (150 min of moderate exercise per week and muscle strengthening exercises on two or more days per week). The exercise program will be customized to the participant and delivered using a web- and mobile-based application. The exercise program will be built for 8 to 12 weeks, depending on study length, with increasing intensity of combined aerobic and resistance exercises over the course of the study. The goal is for participants to perform at least 3 routines per week at their desired location. Total session time may vary by participants depending on number of breaks and rest periods. Exercise routines will combine aerobic, resistance and stretching exercises. In addition, participants will be encouraged to walk daily and increase their daily steps by 500 steps per day every week until they reach 10,000 steps per day. They will be able to monitor their steps with provided pedometers (Fitbit, Garmin, Apple Watch, etc.). Participant’s adherence to their exercise prescription and walking goal will be assessed weekly by the coach.

#### Dietary intervention

The dietary intervention is based on the Mediterranean diet. The meal plan was designed by a Registered Dietitian and follows the 2015 Dietary Guidelines for Americans healthy Mediterranean-style eating pattern (Tables 2 and 3) [31]. Participants will be provided with all meals (3 meals/day) throughout the 8–12-week intervention that are in compliance with the Mediterranean diet in the form of refrigerated packaged meals from a company with the capacity to provide made-to-order meals and deliver them to the participant’s home address. The meals will be heated using conventional equipment found in most households and require no further preparation. Depending on calorie and macronutrient requirements, as assessed by the coach in accordance with participant feedback, snacks that adhere to the Mediterranean meal plan may be added. Snacks will include nutrient dense foods such as whole grains, fruits, nuts, low-fat milk, yogurt, and/or cheese. This nutrition intervention is not designed for participants to lose weight, however with the meal plan that could occur. Participants will be provided with a detailed weekly meal plan and will be asked to keep record of what they eat daily, on paper and/or using RedCap surveys.

**Table 2:** Dietary Guidelines for Americans Mediterranean-Style Meal Pattern

Food Group	Daily Goal^a^
Vegetables	2.5 c-eq.
Fruits	2 c-eq.
Grains	6 oz.-eq.
Dairy	2 c-eq.
Protein	6 oz.-eq.
Oils	24 g

^a^Expressed as cup-equivalents or ounce-equivalence in accordance with the Dietary Guidelines for Americans standard practice [32]

**Table 3:** Example menu day

Meal	Food Group Pattern^a^	Example Menu Items
Breakfast	1 c-eq fruit	1 Medium Apple
	2.8 oz.-eq grains	1 Blueberry Bagel
	1 c-eq dairy	2 Low-fat Cream Cheese Wedges
	1 oz.-eq nuts	1 Snack Pack Mixed Nuts
Lunch	2.5 c-eq vegetables, 1.3 oz.-eq grains, and 5.5 g oils^b^	Pesto Pasta with Sautéed Greens, Carrots, Tomato, and Zucchini Mixed Dish
Dinner	4 oz.-eq protein	Salmon
	2.5 c-eq vegetables, 1.3 oz.-eq grains, and 5.5 g oils^b^	Smoked Paprika Vegetables (sautéed broccoli, carrots, kale, and tomato), and Brown Rice Mixed Dish
	13.5 g oils	1 TB Olive Oil
Snack	1 c-eq fruit	2 Clementines
	0.6 oz.-eq grains	5 Multi-Grain Crackers
	1.1 c-eq dairy	2 Light String Cheese Sticks
	1 oz.-eq protein	3 Slices Deli Turkey Breast

^a^Equivalents are obtained through NDSR and rounded to the nearest tenth

^b^Mixed dish equivalents are based on 7 randomly selected vegetarian line meals from the food vendor

#### Standard of care

Participants in the standard of care group will be provided with dietary and physical activity recommendations from the Dietary Guidelines for Americans at their first study visit and will follow the standard of care practice at AF&GA up to their retrieval procedures. Participants will not be required to wait 8 weeks prior to their retrieval procedures since no intervention is needed. If participants in the standard of care group plan to undergo a second oocyte retrieval cycle, they will be offered to join the intervention group at that time as a cross over design.

#### Compensation and incentives

Participants will be compensated for their time spent at the research facility, their traveling costs and efforts with a monetary value of $50 per visit. Participants randomized to the intervention group will also receive all free meals for a period of 8 to 12 weeks depending on their fertility treatment plan. They will also receive training assistance, coaching, and reimbursement for monthly fees to a gym of their choice up to $50/month for the duration of the intervention. As an incentive to keep up with the exercise intervention, participants will be offered $10/week for meeting their step and exercise goal. A bonus of $80 will be provided to participants who completed all weekly exercise sessions prescribed. Participants in the standard of care group will receive a completion bonus with a monetary value of $200 at their last visit. Total compensation for the standard of care group (all paid by checks or gift cards) will be up to $350 dollars.

### Data collection, management, and analysis

#### Data collection methods

Participants will attend three research study visits at the ACNC, two at the beginning of their fertility procedures and the last within 5 days of their oocyte retrieval procedures. Table 1 and Fig. 1 detail the time schedule of enrollment, participant visits, and the intervention. On average, we anticipate that there will be 8–12 weeks between visit 1 and 3 depending on the participant’s fertility treatment plan. Questionnaires will be administered on paper or via online platforms such as RedCap.

##### Background information and releases

Women who agree to participate will provide background information including their name, address, date of birth, education, number of people living in their household, marital status, and health history. Participants will be required to obtain an exercise release form from their physician at AF&GA prior to the study visit. They will also be required to sign a consent for release of medical information in order to obtain data relating to the clinical results from the fertility procedures as well as pregnancy and delivery outcomes.

##### Anthropometrics and vitals

Anthropometric data will include height, weight, and waist and hip circumferences. All measures will be repeated in duplicate or triplicate in order to ensure measures fall within stated tolerance ranges. Height will be measured to the nearest 0.1 cm with a wall-mounted stadiometer (tolerance of +/− 1 cm). Weight will be measured to the nearest 0.1 kg on a scale that has been tared with clothing the participant is wearing (tolerance of +/− 0.1 kg). Waist and hip circumferences will be measured to the nearest 0.1 cm using a tape measure against bare skin or form fitted shorts (tolerance of +/− 1 cm).

Vitals will include blood pressure, pulse, and temperature. Blood pressure and pulse will be measured with a digital blood pressure monitor twice (with 5 min in between each measurement); a third measurement will be taken if the two measures have a difference of 6 mmHg for systolic or 4 mmHg for diastolic blood pressure. Temperature will be taken upon arrival for each visit with an infrared non-contact forehead thermometer in accordance with COVID-19 screening procedures. Participants with a temperature ≥ 100.4 °F will be asked to reschedule their visit while those with a temperature below 100.4 °F will have it taken again orally with a SureTemp Plus thermometer for data collection purposes.

##### Body composition

Body composition and bone mineral content will be measured using dual energy X-ray absorptiometry (DXA; Hologic Horizon A, Bedford, MA). This technology involves very small amounts of radiation and yields a very important measure of body composition and bone mineralization [32]. Quality control calibrations will be performed each day and subjects will be positioned according to the manufacturer recommendations for the whole body scan while wearing light clothing without metal.

##### Resting and activity energy expenditure

Resting energy expenditure will be measured using indirect calorimetry (MOXUS, AEI technologies, IL) following an overnight fast while wearing a heart rate monitor (Polar, Finland) [33]. The instrument measures the flow of oxygen consumed and the flow of carbon dioxide produced to calculate energy expenditure. The system will be calibrated daily. Activity energy expenditure will be assessed using the ActiGraph 9XLink accelerometer (ActiGraph, Pensacola, FL) worn for 7 days and a previously validated physical activity questionnaire (International Physical Activity Questionnaire) [34].

##### Fitness and leg strength tests

A cardiorespiratory fitness test will be performed on a treadmill following the guidelines for exercise testing from the American College of Sports Medicine. After evaluating the participant at rest and during the 3–5 min warm up, speed and/or incline will be increased in periods until exhaustion. During the entire test, breath composition will be sampled and measured using a metabolic cart (Medgraphics Ultima PFX® system, MGC Diagnostics Corporation, St. Paul, MN, USA). Leg strength will be measured by an isokinetic dynamometer (Humac Norm, Computer Sports Medicine Inc., Stoughton, MA). The Humac dynamometer will be used for testing of right and left legs for flexion (hamstrings) and extension (quadriceps) at certain angular velocities.

##### Biological samples

Blood samples, up to 35 ml, will be collected from the participants at visits 1 and 3 by a trained phlebotomist under overnight fasted conditions. During the entire study, we will collect up to 70 ml total. Blood samples will be centrifuged to separate plasma, serum, and red blood cells. Blood samples will be used to assess glucose, insulin, HOMA-insulin resistance score, interleukin-6, C-Reactive Protein, tumor necrosis factor, leptin, lipid profiles or any other biomarkers or analytes of interest using ELISA, electrochemiluminescence (ECL), colorimetric assays, metabolomics or any other appropriate methodology. Urine and stool collection will be obtained to evaluate metabolites, hormones or bacteria/microbes. Samples will be obtained using sterile collection cups. All biological samples will be aliquoted and stored at − 80 °C until further analyses.

##### Depression and perceived stress

Maternal depression will be evaluated using the Beck Depression Inventory-II, which has been shown to have high reliability and validity [35]. Participants’ level of perceived stress will be evaluated through the administration of the Cohen’s Perceived Stress Scale which is a validated 14-item questionnaire examining the perception of stressful life events and levels of experienced stress over the course of the 4 weeks prior to the completion of the survey [36]. Participants with elevated scores will be provided with mental health resources and referred to their primary care provider.

##### Dietary assessment

Baseline dietary intakes will be assessed using 3-day food records. Dietary data will be evaluated by a trained research assistant for completeness and analyzed with the Nutrition Data System for Research (NDSR, Nutrition Coordinating Center, University of Minnesota, MN). Data entry into this software will be randomly audited (10%) by a trained research assistant to ensure quality control.

##### Oocyte, cumulus cell, and follicular fluid data collection

AF&GA will provide the research team with oocytes (eggs), cumulus cells (cells surrounding the egg), and follicular fluid (liquid surrounding the egg that also contains granulosa cells) that are not needed for the IVF procedures (e.g., too many eggs retrieved). These samples will be collected during the standard oocyte retrieval procedures performed as part of standard care for the participants undergoing fertility treatment. Participants will undergo standard procedures at AF&GA for ovarian stimulation, which include hormonal superovulation and oocyte retrieval 36 h after human gonadotropin injection. Any deviations from the standard clinic procedures will be documented and sensitivity analyses will be performed to determine whether deviations to stimulation protocol yielded in significant differences. It is possible that the research team will not obtain samples in the case that the retrieval yields only enough eggs for the IVF procedures. In this case, only follicular fluid will be obtained. The research team will not obtain samples that could have been fertilized or frozen as agreed with the participant prior to the retrieval procedure. The research team will obtain only samples that are unneeded by the clinic. Thus, no additional treatment or clinical procedures will be required for the participants. The embryologist at the clinic will collect the cumulus cells during the processing of the oocytes. Granulosa cells will be isolated from the follicular fluid by centrifugation at 4000 rpm for 10 min. Follicular fluid samples contaminated with blood will not be used for analysis. Samples will be placed on ice and transported by the research team to the ACNC where they will be stored in messenger RNA lysis buffer and frozen at − 70 °C until processing. Oocytes and cumulus cells will be used to study gene expression while follicular fluid will be used to measure metabolites and hormones that may be influenced by diet and exercise. Both mRNA and genomic DNA will be isolated from individual oocytes as previously described [9]. cDNA libraries will be prepared and fragmented cDNA will be evaluated to determine size distribution of the libraries. Samples will be sequenced using the NextSeq 500 System (Illumina). Individual libraries will be generated and RNA-Seq will be conducted. Sequencing reads from each sample will be trimmed and filtered before being aligned to the human genome (hg19). All data will be analyzed using SeqMonk and R software. Differentially expressed gene expression will be identified using the DESeq2 package which will include multiple testing corrections. Serum and follicular fluid glucose, total cholesterol, and triglyceride levels will be assessed using colorimetric or electrochemiluminescence assays. Serum and follicular fluid insulin, leptin, tumor necrosis factor (TNF)-α, C-reactive protein (CRP), and interleukin-6 will be measured using enzyme-linked immunosorbent or electrochemiluminescence assay kits. Serum lipid profiles—non-esterified fatty acids, high-density lipoprotein, and low-density lipoprotein— will be analyzed by enzymatic methods on a clinical analyzer. All assays will be performed in accordance with the manufacturers’ instructions.

##### Feasibility and acceptability

Feasibility and acceptability will be measured primarily through structured qualitative interviews that will take place after intervention completion. These interviews will be conducted by trained team members in-person or virtually through video conferencing and are expected to last approximately 60 min. They will be audio recorded and transcribed before being coded and analyzed to identify key factors contributing to feasibility and acceptability of the intervention. Compliance with the intervention provides another measure of feasibility and acceptability. Participants in the intervention group will track their daily dietary intake for the duration of the intervention using paper and/or RedCap surveys. These surveys will be reviewed weekly by the health coach for completeness before being analyzed with NDSR (NDSR, Nutrition Coordinating Center, University of Minnesota, MN). To account for deviations from the meal plan, overall dietary compliance will be evaluated by comparing participant’s Healthy Eating Index (HEI) score to that of the standard meal plan. Participants will track their adherence to the exercise intervention (150 min/wk. exercise) using personal training software (FitSW, FitSW Inc., Colorado Springs, CO) and their steps will be continuously monitored by a pedometer (Fitbit, Garmin, Apple Watch, etc.) provided by the research team. Compliance with the exercise prescription (150 min/week) and walking goal (500 extra steps/week) will be assessed weekly by the health coach. Coaches will document the average number of weekly steps and total exercise minutes.

#### Data management

All investigators and research team members will complete and maintain appropriate CITI training. All data will be entered into the ACNC clinical relational database and/or RedCap. The database will be housed on a shared drive that is backed up nightly off-site Monday through Friday. Access to the server is password protected, as is access to the study database. Data and study samples will be labeled with a unique identifier (e.g., MB-101). This identifier will be in no way associated with the participant’s personal information, such as name or date of birth. The key to the coded information, all identifiers, and biological specimens will be destroyed 7 years after the study close date. Data and samples will not be used for future research, either with or without identifiers. Participant names, contact information, and address will be shared with the meal preparation and delivery company. Data and samples will only be accessible by investigators, research assistants, and institutional oversight offices (e.g., IRB).

#### Statistical analyses

Prior to inferential analyses, exploratory data methods will be used to check for potential outliers and aberrant observations and measurements. The following will be tested between the PCOS and no PCOS groups to assess potential differences: fertility diagnosis (male factor, ovulatory disorder, tubal factor, endometriosis or unexplained), pregnancy, live birth, age and race using Mann-Whitney U nonparametric tests for each strata. Follicular fluid and serum metabolites will be analyzed using a one factor Analysis of Covariance (ANCOVA) at four levels because of the potential unbalanced data structure. Pair-wise comparisons using Tukey-Kramer post-hoc tests will be used to identify groups whose means differ significantly while retaining the family-wise error rate at 5%. If the distributional assumptions for ANOVA are not met, and if a suitable data transformation is not found, a Kruskal-Wallis equality-of-populations rank test will be used, followed by Dunn’s test of multiple comparisons. When the design is balanced, two-way ANOVA or non-parametric Friedman test will be used in data analysis. As alternatives, generalized linear models may be also used to test the effects of treatment and presence of PCOS and ANCOVA may also be used to control for additional confounding factors Correlations will be determined using the Pearson Product Moment or the Spearman Rank Order for non-parametric tests. Significance will be set at *p* < 0.05. RNA-seq data will be analyzed using SeqMonk and R software. Gene expression levels will be expressed as raw read counts for differential expression analysis and as log-transformed normalized reads per kilobase per million mapped reads for visualization. Differentially expressed gene expression will be identified using the DESeq2 package and filtered at ± two-fold change and statistical significance with a *p* value ≤0.05. Gene Ontology (GO), transcription factor (TF) target analysis, and pathway analyses will be conducted with DAVID bioinformatics and/or WebGestalt, which include multiple testing corrections. For participants assigned to the standard of care whose fertility treatments were unsuccessful, they will be offered to re-enroll and be directly assigned to the intervention group. The data from these participants will be analyzed separately, using paired t-tests or non-parametric equivalent to evaluate the effect of the intervention (pre and post).

#### Sample size

Based upon existing data in the literature, a power of 0.8, and a *p*-value set at 0.05, with 20 women per group, we will be able to detect a mean difference of at least 0.37 standard deviation units in follicular levels of C-reactive protein and of insulin. In our previous study, we found follicular fluid CRP differences of 3.2 μg/ml (2.7 vs. 5.9 μg/ml, respectively) between normal weight and obese women [12]. Thus, we are confident that this sample size will be sufficient to detect clinically meaningful differences. Based on our experience, we expect that we will be unsuccessful in obtaining a good quality oocyte from approximately 33% of women enrolled. Therefore, we will enroll 30 women in each of the 4 groups (PCOS- standard of care, PCOS-intervention, no PCOS- standard of care, no PCOS-intervention) to assure that we will have at least 20 oocytes per group.

### Monitoring

A Data and Safety Monitoring Plan is in place for this study. Adverse events, enrollment numbers, lost to follow up counts, and withdrawals will be monitored by the Clinical Coordinator. A continuing review will also be completed annually. Any adverse events and deviations will be reported to the IRB and included in the continuing review. Any adverse effects or unanticipated problems will be reported to the study PI, the IRB, and the study sponsor in accordance with IRB policies. At all times, the IRB will be promptly provided with the information from the Clinical Coordinator as needed.

## Discussion

We anticipate that women engaged in the intervention will have a decreased oocyte expression of genes related to inflammation and oxidative stress and lower Indole-3-Proprionic Acid (IPA) content in follicular fluid compared to women who received the standard of care because of evidence demonstrating the positive effects of the Mediterranean diet on fertility rates and pregnancy outcomes [7, 20, 22]. We also expect that women engaged in the intervention will have greater fitness level, higher insulin sensitivity, lower triglycerides, lower blood pressure and lower systemic inflammation at the end of the intervention compared to women in the standard of care group. There is indisputable evidence on the beneficial effects of a Mediterranean diet and physical activity on chronic diseases like diabetes, cardiovascular disease, cancer, and neurodegenerative diseases [19, 37, 38]. Further, when both lifestyle factors are incorporated during the peri-conception period and followed throughout pregnancy greater protections may occur [38]. These findings suggest the importance of individualized interventions, with future RCT’s exploring interventions with both physical activity and Mediterranean-style eating patterns, especially in the pre-conception period to ensure best outcomes throughout pregnancy [38].

One study suggested better Mediterranean diet scores were positively associated with Luteal Phase Deficiency (LPD) after adjusting for age, percentage body fat, and total energy intake [39]. LPD may contribute to infertility and early miscarriage; one study suggested that diet quality may be associated with LPD among healthy eumenorrheic women, therefore further research is warranted to elucidate how dietary factors, such as Mediterranean style eating patterns, may influence LPD and other causes of infertility [39]. Emerging evidence suggests that a RCT with PA may improve pregnancy rates in women with reproductive health problems [11]. Comparative studies indicate that PA intervention may be as effective as other commonly used clinical intervention strategies for improving reproductive health outcomes. Preliminary findings suggest that PA may be an affordable and feasible alternative or complementary therapy to fertility treatments, while the type, intensity, frequency, and duration of optimal PA intervention, and the role of PA independent of weight loss, remain unclear [11].

Therefore, the need to further investigate how lifestyle factors including exercise, diet, and behavioral modifications affect fertility is imperative and more emphasis should be placed on physiological consequences of an unhealthy lifestyle during the critical peri-conception window. Future collaboration between research institutions, clinical practice, and communities will enable the implementation of more evidence-based recommendations and interventions such as counseling to improve dietary behaviors or nutritional programs aimed at reproductive aged populations and at risk groups like those undergoing fertility treatments [40]. Moreover, new evidence through larger more in-depth studies will support the development of lifestyle interventions to improve the health of mothers and their offspring from the earliest moment in life [41]. Thus, given the potential of the intervention being tested for adoption into clinical practice, it may help women suffering with overweight or obesity and infertility.

### Strengths

This is a robustly designed RCT that uses valid and reliable measures with standardized procedures and quality control measures. Strengths of this study lie with ease of compliance during the 8–12 week exercise and nutrition intervention, with the majority of food shipped directly to the participant, and all exercises being self-directed and accessed through an application on the participant’s phone or computer. In addition, the participant’s engagement with health coaches will increase adherence and compliance.

### Contingencies

Recruitment may be challenging because we will be enrolling women who want to conceive as soon as possible and who may choose to immediately attempt in vitro fertilization rather than wait. However, these women will have a high motivation to achieve the best outcomes for their treatments, which are known to be improved by increasing physical activity and adhering to a healthy diet.

Sedentary women with obesity may also be reluctant to participate in PA or implement dietary changes. To counter compliance issues, participants will have twice-weekly phone contact with the trained professionals at ACNC. We believe participants randomized to the intervention group will have a higher rate of participation due to their urgency to conceive and lack of pregnancy symptoms that can make it more difficult to engage in exercise routines. While we will track diet compliance through daily food diaries, this approach cannot fully capture non-compliance with the dietary intervention. For this reason, we will collect red blood cells for analysis of enrichment in PUFAs in the intervention groups. Stool will also be collected to enable a secondary measure of microbiome differences across groups. We anticipate that intervention participants, whose diet will contain significantly more fiber, fruits, and vegetables compared to the typical American diet, should be readily distinguishable from the control group.

The research team at the ACNC has experience in all aspects of the proposed study. Therefore, minimal problems are expected regarding the techniques proposed. One potential concern is participant non-compliance or drop out. Accordingly, we anticipated this issue in our sample size calculation. Absence of an effect of the intervention is also a concern; in this case, a study with a longer intervention or an additional exercise intervention would be explored for future studies.

## Conclusion

Our goal is to assess the feasibility and efficacy of a peri-conception nutrition and exercise intervention on mitigating obesity-associated changes in oocyte gene expression profiles and follicular fluid metabolites. This is possible through a partnership between AF&GA, who will provide participants and samples, and the ACNC who will carry out the intervention, collect and evaluate data, and disseminate findings. This study addresses a significant scientific question on how to effectively engage women undergoing fertility treatments and will provide valuable insights on metabolic programing that occurs during the peri-conception period.

## Data Availability

Not applicable.

## Abbreviations

IVF: In-vitro fertilization
PA: Physical Activity
CRP: C-Reactive Protein
PCOS: Polycystic Ovary Syndrome
BMI: Body Mass Index
ACNC: Arkansas Children’s Nutrition Center
IRB: Institutional Review Board
DXA: Dual-Energy X-ray Absorptiometry
RER: Respiratory Exchange Ratio
NDSR: Nutrition Data System for Research
ECL: Electrochemiluminescence
ANOVA: Analysis of Variance
ANCOVA: Analysis of Covariance
GO: Gene Ontology
TF: Transcription Factor
LPD: Luteal Phase Deficiency
AF&GA: Arkansas Fertility and Gynecology Associates Clinic
IPA: Indole-3-Proprionic Acid
TNF: Tumor Necrosis Factor
HEI: Healthy Eating Index
DOHaD: Developmental Origins of Health and Disease
